# Gene expression of human endometrial L-selectin ligand in relation to the phases of the natural menstrual cycle

**DOI:** 10.1038/s41598-018-19911-z

**Published:** 2018-01-23

**Authors:** Tsung-Hsuan Lai, Fung-Wei Chang, Jun-Jie Lin, Qing-Dong Ling

**Affiliations:** 1 Department of Obstetrics and Gynecology, Cathay General Hospital, Taipei, 10693 Taiwan; 2 School of Medicine, Fu Jen Catholic University, New Taipei City, 24205 Taiwan; 3 Institute of Systems Biology and Bioinformatics, National Central University, Taoyuan City, 32001 Taiwan; 4 Department of Obstetrics and Gynecology, Tri-Service General Hospital, National Defense Medical Center, Taipei, 11490 Taiwan; 5 Cathay Medical Research Institute, Cathay General Hospital, New Taipei City, 22174 Taiwan

## Abstract

This study investigates peptide components of L-selectin ligand (LSL) and their gene expressions in human endometrium during the natural menstrual cycle. We recruited 41 endometrial samples from reproductive-aged women with leiomyoma and undergoing hysterectomy and 11 endometrial samples from menopausal women as controls. Immunohistochemistry revealed strong MECA-79 expression from the early through the mid-secretory phase and low expression in menopausal endometrium. Five peptide components of LSL were detected in reproductive and menopausal endometrium by one-step quantitative RT-PCR: podocalyxin, endomucin, nepmucin, GlyCAM-1, and CD34. Endomucin differed significantly between the proliferative and early-secretory phases. CHST2 and CHST4 genes (which are involved in the generation of LSL epitopes) were expressed without significant differences among phases. The gene expression of progesterone receptor decreased from the proliferative to the late-secretory phase, and the difference was significant. However, estrogen receptor α expression showed stability among phases. The significant expression of endomucin between the proliferative and early-secretory phases might play a vital role in endometrial receptivity. Further studies are needed to investigate the factors that regulate the expression of endomucin and other LSL peptide components in different phases of the menstrual cycle.

## Introduction

The endometrium is a highly differential tissue that develops with the changes of the menstrual cycle and supports the dynamic events required to establish and maintain pregnancy. In humans, increasing evidence suggests that the preparation of the endometrium for embryo implantation, although dependent on adequate hormonal stimulation, also requires interaction between the blastocyst and the endometrium^1^. This interaction is mediated by a variety of factors that are produced and secreted by both the endometrium and the blastocyst^2^. Evidence indicates that the initial attachment of an embryo to the endometrium depends on the binding of L-selectin expressed by the trophoblast and oligosaccharide-based ligands expressed by the endometrium^3^. Therefore, the interactions between L-selectins and their ligands in the endometrium may act as a bridge for the initial attachment during implantation^4^, which supports the concept that the expression of L-selectin ligands(LSLs) might reflect the receptivity of the endometrium.

L-selectin ligands in high endothelial venules (HEVs) of lymph nodes are glycoproteins that serve as addressin (address signals) for homing and support of lymphocyte extravasation^5^. The ligands include CD34, podocalyxin, GlyCAM-1, endomucin, and nepmucin in mouse. All have mucin-like polypeptide structures with sulfated O-linked glycans^6^. In our previous study, we used MECA-79 antibody, which recognizes a high-affinity LSL carbohydrate epitope, to detect the expression of LSLs in human endometrium. We found that LSLs were expressed differentially at the different phases in the natural cycle. They were upregulated at the secretory phase and downregulated at the proliferative phase. The expression of LSLs reached the highest level at the mid-secretory phase, which coincides with the implantation window period^7^.

However, thus far, there are no studies exploring the gene expression patterns of LSLs in human endometrium throughout the menstrual cycle. One of the main reasons is the difficulty of obtaining sufficient endometrial specimens from human subjects. Immunoblotting revealed at least four components of LSL expression in human endometrium^8,9^. However, little information is available on these LSL peptide components. Fundamental questions about the types of components and gene expression patterns at different phases of the natural cycle are unresolved. For example, it is unclear whether the types of LSL peptide components in human endometrium are the same as that in the HEVs of mouse lymph nodes. Another issue is what their roles are in relation to endometrial receptivity in the human endometrium. The gene expression patterns of LSL peptide components in the different phases of the natural menstrual cycle have also been unclear.

One of the drawbacks of previous studies is the uncertainty regarding whether the expression in one biopsy is truly representative of what happens throughout the whole cycle. Therefore, we conduct this study to investigate the gene expression patterns of LSL peptide components in human endometrium at different phases of the natural menstrual cycle.

## Results

Table 1 summarizes the demographic data of the subjects during the four phases of the menstrual cycle. There were 41 endometrial biopsies, which included 11 from the proliferative phase (days 7 to 14), 9 from the early-secretory phase (days 15 to 19), 10 from the mid-secretory phase (days 20 to 24), and 11 from the late-secretory phase (days ≧ 25). In addition, 11 endometrial samples were obtained as controls from menopausal women with uterine prolapse who underwent vaginal hysterectomy. The mean ages of cases in the proliferative, early-secretory, mid-secretory, and late-secretory phase were 44.8 ± 4.0, 45.4 ± 4.4, 46.1 ± 4.9, and 47.9 ± 3.2 years, respectively. The average BMI of the cases was 24.7 ± 5.7 kg/m^2^ for the proliferative phase, 21.7 ± 1.4 kg/m^2^ for the early-secretory phase, 24.0 ± 3.4 kg/m^2^ for the mid-secretory phase and 25.1 ± 2.6 kg/m^2^ for the late-secretory phase. The mean age and BMI of the patients were not significantly different between phases (*P* > 0.05) (see supplementary Table 1).

**Table 1:** Demographic data of the subjects at different phases of the menstrual cycle.

Phases	Proliferative	Early-secretory	Mid-secretory	Late-secretory	*P* value^1^
	(day 7–14)	(day 15–19)	(day 20–24)	(day ≧25)	
No. of subjects	11	9	10	11	
Mean ± (SE) age (y)	44.8 ± 4.0	45.4 ± 4.4	46.1 ± 4.9	47.9 ± 3.2	0.231
Mean ± (SE) BMI (kg/m^2^)	24.7 ± 5.7	21.7 ± 1.4	24.0 ± 3.4	25.1 ± 2.6	0.123

^1^*p* value was K-W test between four phases in leiomyoma. No significant differences were observed between the groups (P > 0.05).

**Figure 1:**
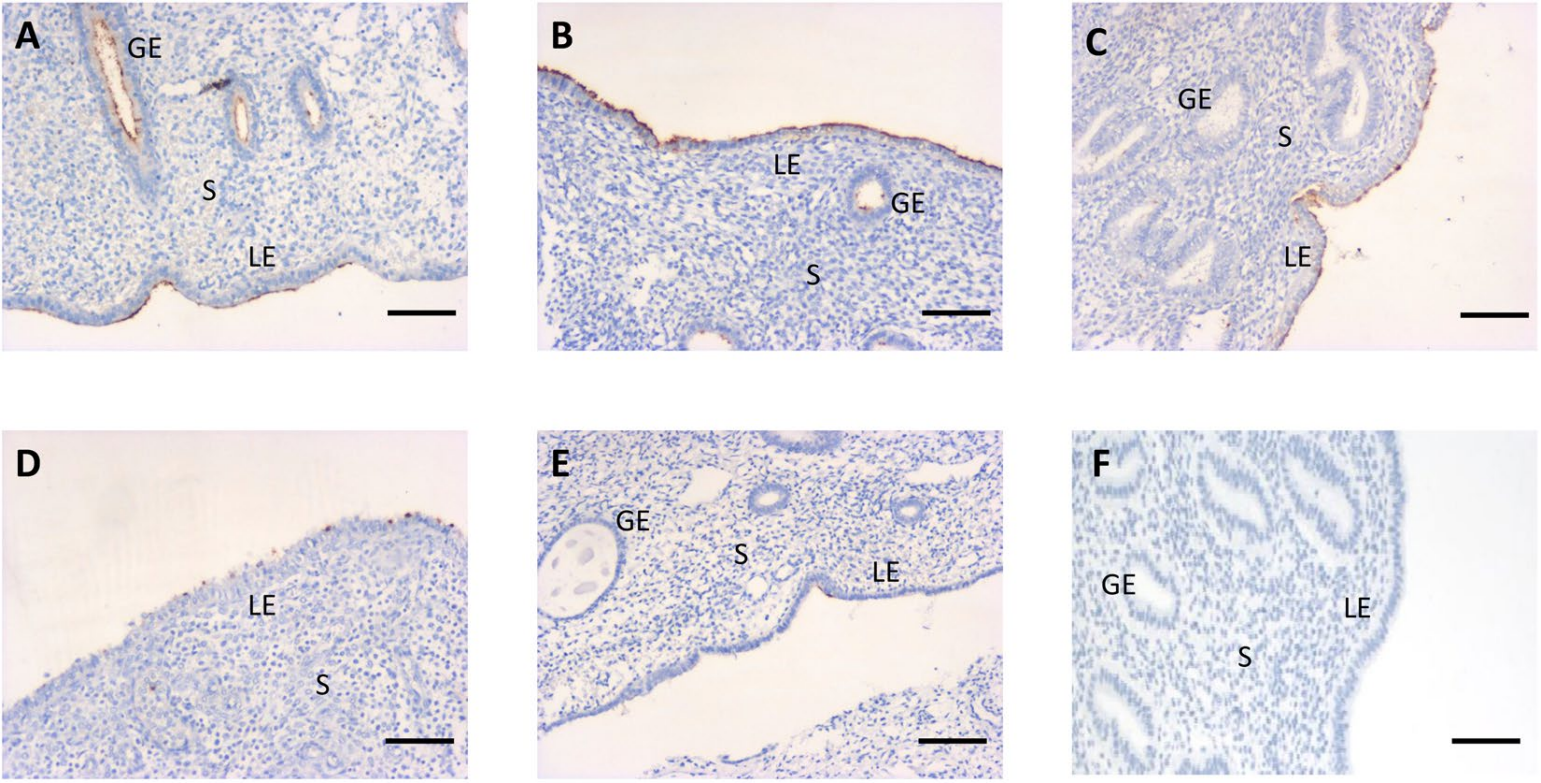
Immunohistochemical staining with MECA-79 in human endometrium for LSL expressed in patients with leiomyoma. Staining was patchy and much less intense during the proliferative phase than during the secretory phase. Weak staining intensity was found in menopausal endometrium. Endometrial tissues at (**A**) proliferative (cycle days 7–14), (**B**) early-secretory (cycle days 15–19), (**C**) mid-secretory (cycle days 20–24), (**D**) late-secretory (cycle days ≧ 25) phases, (**E**) menopause, and (**F**) negative control. LE = luminal epithelium; GE = glandular epithelium; S = stroma. Original magnification 40×. Scale bar = 50 μm.

Immunohistochemistry showed that LSLs were expressed in the luminal and glandular epithelium of the endometrium in all four phases. The MECA-79 intensity of LSLs varied between phases and was strongly expressed at the early and mid-secretory phases (Fig. 1). Interestingly, weak expression of LSLs was also found in menopausal endometrium (Fig. 1).

**Figure 2:**
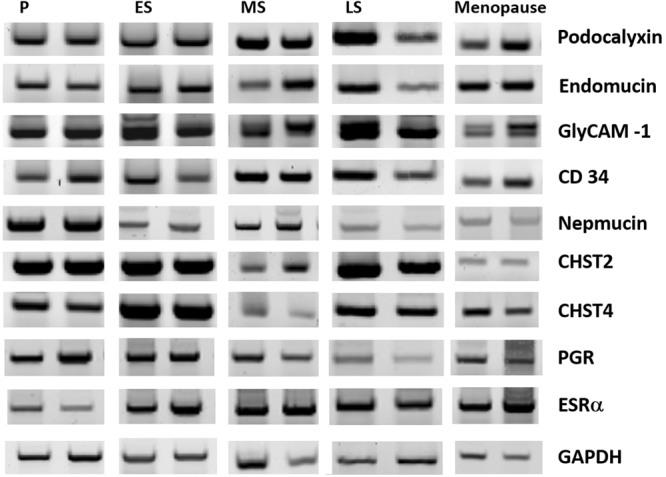
Gel electrophoresis of RT-PCR products during the four phases of the menstrual cycle. Cropped gels of RT-PCR products showed that the mRNA of the LSL peptide components podocalyxin, endomucin, GlyCAM-1, CD34, and nepmucin were differentially expressed in the four phases of the menstrual cycle and menopausal endometrium. Gene expressions of sulfotransferases (CHST2 and CHST4), ESRα and PGR were also found in the four phases of the menstrual cycle and menopausal endometrium. The mRNA levels were normalized to GAPDH mRNA levels. P: proliferative phase; ES: early-secretory phase; MS: mid-secretory phase; LS: late-secretory phase. (Examples of full-length gels are included in a supplementary Figure 2A and B).

**Figure 3:**
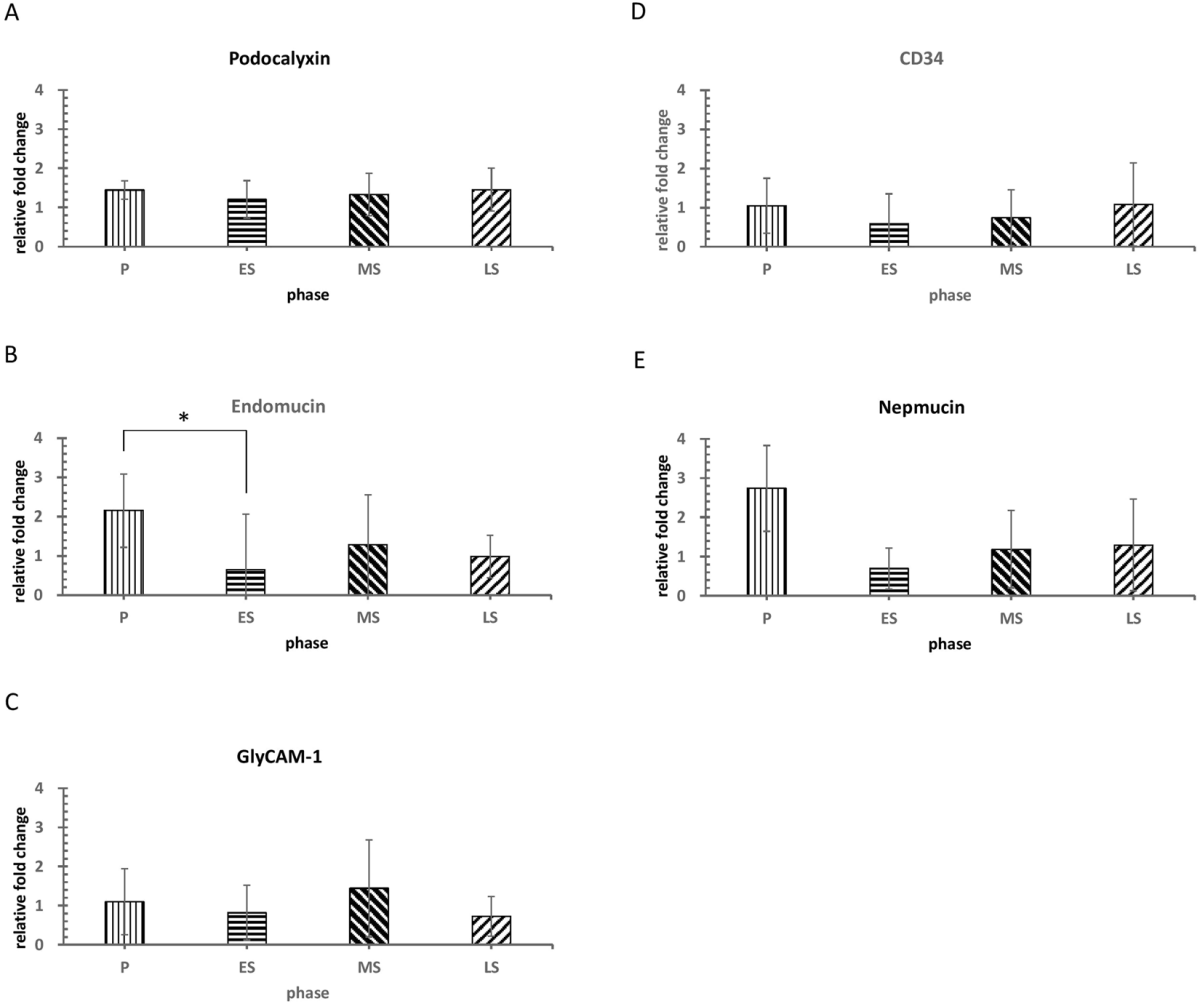
The expression patterns of LSL peptide component genes in the four phases of the menstrual cycle. The gene expression of LSL peptide components revealed variation among the four phases. The relative fold changes of endomucin, nepmucin, and CD34 are higher in the proliferative phase (**B**, **C** and **D**). There is a significant difference in relative fold changes in endomucin between the proliferative and early secretory phases (P < 0.05) (**B**). Results are expressed as mean ± SE. Statistical significance was assessed by the Kruskal-Wallis test (*p < 0.05). P: proliferative phase; ES: early-secretory phase; MS: mid-secretory phase; LS: late-secretory phase.

We applied a one-step quantitative RT-PCR method to examine mRNA expression levels. Five types of LSL peptide components were detected in the reproductive and menopausal endometrium: podocalyxin, endomucin, nepmucin, GlyCAM-1, and CD34 (Fig. 2) (see supplementary Fig. 1A and B). The expression patterns of LSL genes revealed variation among the four phases. The relative fold changes of endomucin, nepmucin, and CD34 are higher in the proliferative phase. The relative fold changes in endomucin are significantly different between the proliferative and early-secretory phases (P < 0.05) (Fig. 3). A Post-hoc Mann-Whitney U test was performed for comparing the difference in endomucin expression between the proliferative and early-secretory phases. A significant difference (P = 0.037 < 0.05) between the two groups, detailed in supplementary Figure 2, was determined.

**Figure 4:**
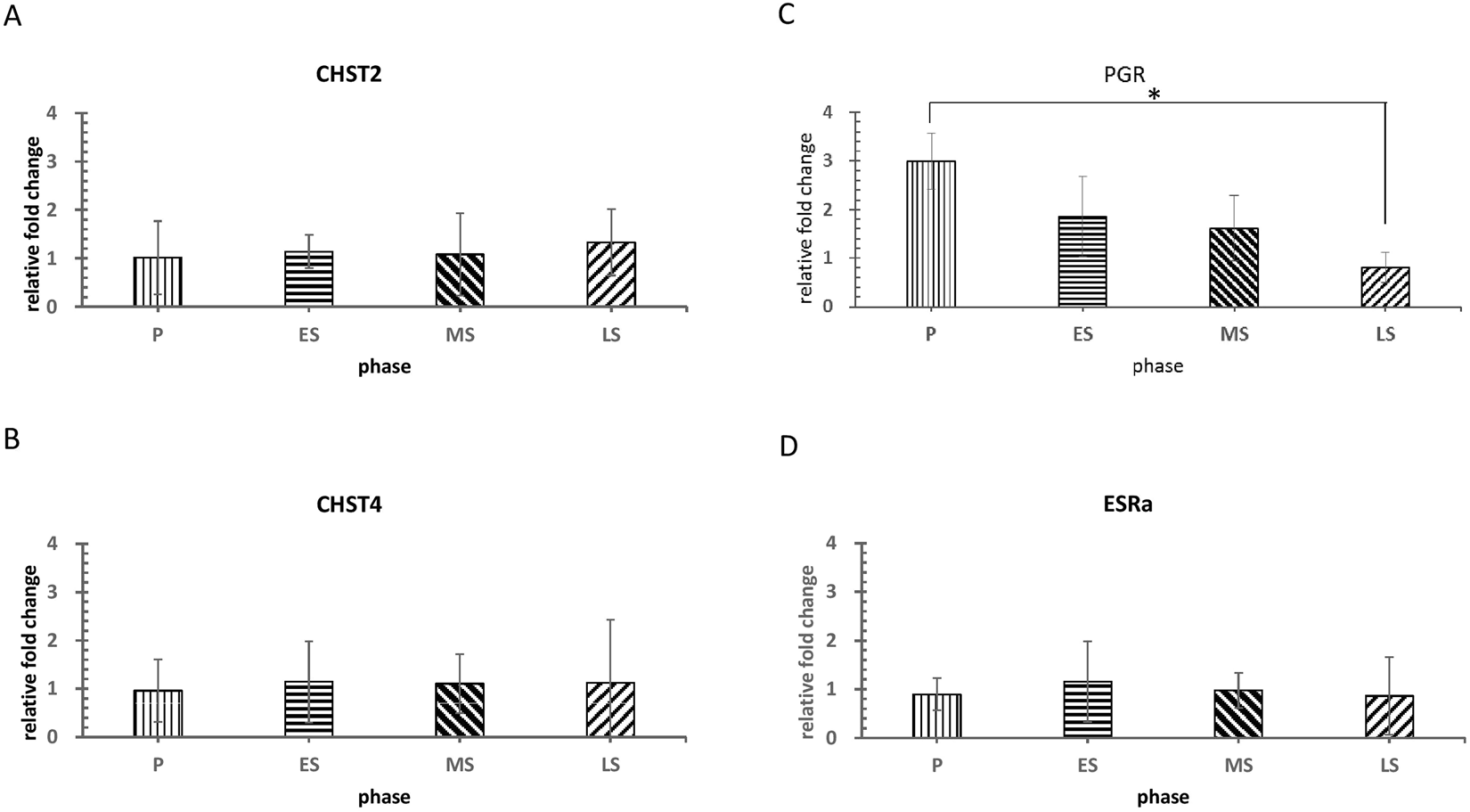
The expression patterns of CHST2, CHST4, ESRα and PGR genes in the four phases of the menstrual cycle. The gene expression of CHST2 and CHST4 was steady among the four phases (**A** and **B**). ESRα also revealed relatively stable expression among the four phases (**D**). PGR expression gradually decreased from the proliferative to the late-secretory phase. There is a significant difference between proliferative and late-secretory phases (P < 0.05) (**C**). Results are expressed as mean ± SE. Statistical significance was assessed by the Kruskal-Wallis test (*p < 0.05). CHST2: carbohydrate sulfotransferase 2; CHST4: carbohydrate sulfotransferase 4; ESRα: estrogen receptor α; PGR: progesterone receptor.

The gene expressions of CHST2 and CHST4, which are the key enzymes for the generation of LSL epitopes, were steady among the four phases. Estrogen receptor α (ESRα) expression was also relatively stable without significant differences among the four phases. However, the gene expression of progesterone receptor (PGR) showed a significant, gradual decrease from the proliferative to the late-secretory phase (P < 0.05) (Fig. 4).

## Discussion

To the best of our knowledge, this is the first time to find the five types of LSL peptide components including podocalyxin, endomucin, nepmucin, GlyCAM-1, and CD34 in human endometrium throughout the natural menstrual cycle (Fig. 2). Interestingly, these five types are the same as those found in the HEVs of mouse lymph nodes in previous studies^5,10–13^. Furthermore, we also found that these five components are present in the menopausal endometrium (Fig. 2). Our results indicate that LSLs are usually present in human endometrium, regardless of whether the woman is at reproductive or menopausal age. This implies that these LSLs might serve as basic adhesion molecules in the human endometrium, which are not only largely induced during the window of embryo implantation, but also differentially expressed in non-pregnant and menopausal endometrium.

The gene expression patterns of LSL peptide components throughout the natural cycle are quite different from the expression pattern of MECA-79 epitope on the oligosaccharide part of LSL in our previous study^7^. The gene expression patterns of LSL peptide components revealed variation between the phases of the natural cycle. The relative fold changes of endomucin, CD34, and nepmucin are higher at the proliferative phase. The relative fold changes in the endomucin were significantly different between the proliferative and early-secretory phases (P < 0.05) (Fig. 3). However, in our previous study, the expression of MECA-79 epitope on LSLs was upregulated during the luteal phase and downregulated during the proliferative phase. LSL reached the highest expression at the early to mid-secretory phases, which coincides with the window of implantation^7^. This suggests that the assembly of LSLs involves in the production of LSL peptide components beginning in the proliferative phase and then post-translational modification with oligosaccharides mainly in the early and mid-secretory phases.

We found that endomucin is the only LSL peptide component with significantly different relative fold changes between the proliferative and early-secretory phases (P < 0.05) (Fig. 3). The expression pattern throughout the cycle is almost consistent with the cyclic change of serum estrogen in the natural menstrual cycle. This raises a concern of whether cyclic-changed estrogen regulates the production of endomucin. Further studies are needed to elucidate this issue. Human endomucin is a 261-aa, 27.5-kDa protein with a transmembrane sequence and multiple glycosylation sites. Human endomucin is abundant in highly vascular tissues such as the heart, kidney, and lung^14^. Endomucin was specifically detected on endothelial cells of the blood and lymphatic vessels of most human tissues. In addition, endomucin was found in the epithelium of the epidermis as well as the epithelial and myoepithelial cells of the eccrine and apocrine glands in the skin^15^. Endomucin isolated from lymphatic tissue carries the PNAd-specific carbohydrate epitope MECA-79, which demonstrates that endomucin belongs to the group of glycoproteins defined as PNAds (addressin). Little is known about this LSL peptide component in human endometrium. A previous study showed that endomucin is a sialomucin with adhesive or anti-adhesive activity, depending on the way it is glycosylated^15^. Moreover, endomucin is a transmembrane protein, which may be related to the post-adhesion signaling pathway in endometrial cells. It could also be involved in endometrial remodeling and promote embryo implantation. Therefore, significant gene expression of endomucin between the proliferative and early-secretory phases suggests that endomucin might play a vital role in endometrial receptivity.

Sulfotransferases are involved in post-translational modifications of PNAd and have been identified in the HEVs of lymph nodes. Five members of the sulfotransferase (GlcNAc6STs) family have been cloned in humans, of which four have orthologues in mice^16^. Among the sulfotransferases, GlcNAc6ST-1 (gene: CHST2) and GlcNAc6ST-2 (gene: CHST4) play dominant roles in LSL biosynthesis^6^. In our study, we found that CHST2 and CHST4 gene expressions were steady without significant changes throughout the menstrual cycle (Fig. 4). The result suggests that CHST2 and CHST4 are not regulated directly by cyclic-changed estrogen or progesterone during the menstrual cycle.

Estrogen and progesterone play important roles in the regulation of cyclic changes in the human endometrium via ESR and PGRs. Two isoforms of estrogen receptors are known: ESRα and ERβ. These receptors are products of different genes but ESRα is predominantly required for estrogen effects on endometrial proliferation and differentiation^17,18^. We investigated ESRα gene distribution in human endometrium, but the results revealed that ESRα gene expression has no significant difference and little correlation with LSLs between the phases of the menstrual cycle (Fig. 4). The mRNA expression of ESRα throughout the menstrual cycle in this study differed from previous studies. A study reported that ESRα mRNA expression was more prominent than that of ESRβ in all cell types throughout the menstrual cycle especially during proliferative phase^18^. In the secretory phase, both the ESRα and ESRβ mRNA expression was relatively weaker^19^. Taken together, ESRα mRNA expression was stronger during proliferative phase and relatively weaker during secretory phase. However, fewer samples may potentially limit the power of previous studies. More clinical samples are needed to make the final conclusion.

The PGR has two isoforms, PGRα and PGRβ, which are derived from the same gene^20,21^. It is well known that estrogen upregulates the PGR in both the epithelium and stroma of the endometrium by direct actions in the mouse^22^. Our results showed that PGR gradually decreased from the proliferative to the late-secretory phase, and the difference was significant (P < 0.05) (Fig. 4). This suggests that PGR expression may be regulated by estrogen because the decreased pattern of PGR expression throughout the cycle is consistent with the cyclic change of estrogen in the natural menstrual cycle. However, our data do not support any correlation of gene distribution among ESRα, PGR, and LSLs. Further studies *in vitro* are needed to investigate the factors that regulate the distribution of LSLs at different phases of the menstrual cycle.

Although the patterns of ESR and PGRs in the functional endometrial compartments have been described^23^, few studies focus on the mRNA expression patterns throughout the natural menstrual cycle. The patterns of ESR and PGRs throughout the menstrual cycles was studied using immunohistochemistry in previous studies^24^. PGR expression in endometrial glandular epithelium reaches a maximum in the late proliferative and early secretory phases followed by a decrease to various degrees depending on the cell types in the late secretory phase. In contrast, ESR expression reaches a maximum in the late proliferative phase in all types of endometrial and myometrial cells^24,25^. The concentration is greatest in the glandular epithelium. During the early secretory phase, ESR expression declines, followed by an increase in the mid and late secretory phases^26,27^. These changes reflect the cyclic changes in estradiol and progesterone. However, Lessey B. A. *et al*. reported that certain types of uterine receptivity defects such as endometriosis, luteal phase defect and polycystic ovarian syndrome may be caused by the loss of appropriate ERα down-regulation in endometrial epithelial cells during the mid-secretory phase, leading to defects in uterine receptivity^28^. In our study, we explored the mRNA expression patterns of ESRα and PGR throughout the menstrual cycle using RT-PCR. We found that the mRNA expression pattern of PGR is similar with the pattern of PGR protein level detected using immunostaining. However, our study showed the mRNA expression of ESRα differs from previous studies, we think the results suggest autocrine and paracrine regulation may influence the availability of ESRα and PGR. Further studies on the mechanism of local regulation on the expressions of ESRα and PGR using animal models and *in vitro* should be conducted in the future.

So far, the real mechanisms or signaling pathways related to the hormonal regulation of LSL peptide components in human endometrium are unknown. Some potential pathways involved in the interaction of L-selectin and its ligands was found in vascular inflammation, immune response and cancer metastasis. By interaction with LSL, rolling leukocytes transduce signals which include serial activation (tyrosine phosphorylation) of Src family kinases (SFKs) and recruitment of adaptors that convert integrin αLβ2 to intermediate-affinity confirmation, which decrease rolling velocities^29^. There are two pathways involved in L-selectin and LSL signaling cascade in neutrophils. One pathway involves serial activation of phospholipase Cγ, p38MAPK, and GTPase Rap1a^30,31^. The other pathway may involve activation of PI3Kγ and Rac1 to recruit talin1^31,32^. In the present study, endomucin is the only LSL peptide component with significant difference of gene expression in human endometrium. Previous studies showed that endomucin prevents leukocyte contact with adhesion molecules in non-inflamed tissues and that downregulation of endomucin by TNFα stimulation is critical to facilitate adhesion of leukocytes into inflamed tissues. A possible mechanism of endomucin downregulation by TNFα is likely to involve binding to the membrane receptors TNF-R55 and TNF-R75^33^. The GATA2 binding region in promoter region of endomucin gene could be a target by transcriptional regulation^34^. In addition, endomucin as a potent regulator of angiogenesis inhibits VEGF-induced endothelial cell migration, growth, and morphogenesis by decreased phosphor-VEGFR2, phosphor-ERK1/2 and phosphor-p38-MAPK level^35^. However, whether the regulation of endomucin in human endometrium is similar with that in vascular endothelium? Further studies are needed to elucidate this issue.

This study has certain limitations that need to be addressed. The small number of samples could potentially limit the power of the study. Furthermore, the heterogeneity of the samples in each phase of the menstrual cycle could impact the results. It is also difficult to obtain blood samples for each patient to test the serum level of estrogen and progesterone at different phases of the menstrual cycle. Finally, because of ethical considerations, we used the endometrial samples of uteruses with leiomyoma instead of normal uteruses, which could not completely represent the actual natural cycle. Although we excluded the endometrial samples of submucosal leiomyoma in the study, paracrine signaling such as TGF-β3 from intramural leiomyomas to the endometrium may contribute to diminish endometrial receptivity. Interestingly, the decreased expression of HOXA 10 and HOXA 11 genes was seen globally throughout the endometrial cavity, and not exclusively in the case of submucosal leiomyoma^36^. A RT-PCR analysis with a one-step RT-PCR method was performed for the analysis of LSL gene expression. The one-step RT-PCR was utilized for minimizing experimental variation by containing all of the enzymatic reactions in a single environment. The advantages of one-step RT-PCR is that it is quicker to set up, less expensive to use, and involves less handling of samples. Therefore, this technique can eliminate pipetting errors, contaminations, and other sources of errors^37^. However, the starting RNA templates used in one-step PCR are prone to degradation, therefore, it is not recommended for analyses that requires repeated assays from the same sample. In addition, one-step RT-PCR is reported to be less accurate when compared to real-time RT-PCR. Furthermore, real-time PCR is more sensitive and displays a greater dynamic range than one-step RT-PCR^38^. However, the extreme sensitivity of real-time RT-PCR can be a double-edged sword because even the slightest DNA contamination can lead to undesirable results^39^. In this study, one-step RT-PCR was utilized for investigating gene expression patterns of LSL peptide components in human endometrium at different phases during regular menstrual cycles, and for determining the types of LSL peptide components in human endometrium, where accurate quantification each gene dosage is not necessary.

Further study on the LSL system is necessary to determine its role in endometrial receptivity. An array study of the adhesion molecules for endometrium could be done to find out the potential adhesion markers related to endometrial receptivity. In addition, a study on post-adhesion signaling pathways of LSL in the endometrium is needed. Finally, a study on the post-translational process of LSL would be very important for uncovering the functions of LSL in endometrial receptivity.

## Conclusion

In this study, five types of LSL peptide components were detected in human endometrium: podocalyxin, endomucin, nepmucin, GlyCAM-1, and CD34. The significant gene expression of endomucin between the proliferative and the early-secretory phases might highlight its potential importance in endometrial receptivity. Further studies are needed to investigate the factors that regulate the expression of endomucin and other LSL peptide components in different phases of the menstrual cycle.

## Methods

All procedures conformed to the Declaration of Helsinki for research involving human subjects. The Institutional Review Board of Cathay General Hospital approved the use of human specimens (*CGHIRB No.:* CT9681). Formal informed consent was obtained from all patients before the time of sample collection into the study. The medical records of all patients were reviewed retrospectively. This is an observational study with experimental analysis on the gene expression patterns of LSL peptide components in human endometrium at different phases of the natural menstrual cycle.

### Sample collection

Endometrium tissue samples were collected from women with leiomyoma and undergoing hysterectomy from Aug 2008 to July 2009. There were 41 endometrial biopsies, which included 11 from the proliferative phase (days 7 to 14), 9 from the early-secretory phase (days 15 to 19), 10 from the mid-secretory phase (days 20 to 24), and 11 from the late-secretory phase (days ≧ 25). In addition, 11 endometrial samples were obtained as controls from menopausal women with uterine prolapse and undergoing vaginal hysterectomy. Tissues samples were frozen at −80 °C until RNA isolation.

The inclusion criteria of the participants were as follows: 1) age ranging from 35 to 50 years; 2) regular menstrual cycle (28 to 35 days); 3) body mass index (BMI) less than 28; 4) no hormone therapy at least 2 months before surgery; 5) no known gynecological diseases, such as pelvic inflammatory disease (PID), cancers, endometrial hyperplasia, or endometrial polyp; 6) no sexually transmitted diseases (STD), such as HIV, hepatitis B and C, chlamydia, gonorrhea, mycoplasma, syphilis, or cytomegalovirus. Patients who had 1) STDs, 2) PID, 3) pregnancy, 4) gynecological cancers, 5) BMI > 28, 6) coagulopathy, 7) psychological diseases, or 8) submucosal leiomyoma were excluded from the study.

### Immunohistochemistry

Portions of the obtained tissues were fixed in buffered formalin and evaluated by a pathologist experienced in endometrial dating. Separate dating of the luminal and glandular epithelium was done according to the criteria published by Noyes *et al*.^40^. A second portion of each biopsy sample was used to investigate the expression of LSL by immunohistochemistry. The expression of LSLs was examined by immunolocalization with a rat monoclonal antibody MECA-79 (BD Biosciences, San Jose, CA) that recognizes a high-affinity LSL carbohydrate epitope containing SO3 → 6GlcNAc^41^, and the assay was performed as previously described in another literature^7,9^. Briefly, the samples were fixed in 10% buffered formaldehyde for 24 hours, embedded in paraffin, and sections of paraffin with 3 to 4 mm of thickness was prepared on positively charged slides prior to the immunohistochemical analyses. The sections were dewaxed with xylene, followed by descending grades of methanol to distilled water solution, and pretreated with Citra Buffer (Vector H3300, Vector Laboratories, Burlingame, CA) in a steamer (HA900; Black & Decker, Hampstead, MD) at 90° for 20 min. The tissue sections were then labeled with the rat antihuman LSL monoclonal antibody (MECA-79) at a concentration of 3.3 μg/mL with dilution of 1:30 in phosphate-buffered saline (PBS). Positive and negative (no antibody) controls were established by using a section of tonsil and a section of endometrium tissue, respectively. For antigen retrieval, the slides were incubated in CC1 buffer (Ventana) for an hour on heated plates at 100 °C with a Benchmark XT processor. The primary antibody incubation was performed for 32 min at a dilution 1:30 at 37 °C. Positive binding of MECA-79 was detected by a biotinylated rabbit antimouse secondary antibody (at a dilution of 1:800 with PBS), which cross-reacts to the rat primary antibody. After the hybridization of primary and secondary antibodies the HRP conjugated avidin-biotin perioxidase (ABC) complex was analyzed by a Ventana DAB Detection Kit (Ventana-Biotek Solutions Inc., Tucson, AZ). The slides were counterstained with hematoxylin, dehydrated, cleared, mounted in DPX mountant, and evaluated using an Eclipse 80i optical microscope (Nikon, Tokyo, Japan).

### Reverse-Transcription Polymerase Chain Reaction (RT-PCR)

The mRNA expressions of LSL genes were analyzed using one-step quantitative RT-PCR for the five LSL peptide components found in HEVs of the lymph node: podocalyxin, endomucin, nepmucin, GlyCAM-1, and CD34. There are mainly two HEV-expressed sulfotransferases involved in the generation of LSL epitopes and LSL activity: GlcNAc6ST-1 (gene: CHST2) and GlcNAc6ST-2 (gene: CHST4)^5,6^. One-step quantitative RT-PCR was also performed to analyze the CHST2 and CHST4 expression. In addition, to investigate the relationship of expression patterns among LSL genes, ESRα and PGR, one-step quantitative RT-PCR was also performed to survey their patterns during the four phases of the menstrual cycle.

Total RNA was isolated by using the 3-*Zol* reagent (MDBio, Taipei, Taiwan) methods, and the RNA sample was treated with DNaseI (Promega, Madison, WI) to remove traces of genomic DNA. To assure optimal DnaseI activity, the buffer conditions in the RNA solution were adjusted accordingly. RNA absorbance at 260 nm was measured by using a spectrophotometer to obtain a yield in micrograms per micro liter (μg/μL). Quantitative RT-PCR was performed with 1 μg of total RNA from the endometrial specimens. cDNA was synthesized and amplified by using a Titanium One Step RT-PCR Kit (BD Biosciences Clonetech, Palo Alto, CA). The cDNA levels of the genes of interest were measured using specific primer pairs, which are described in details in Table 2. Glyceraldehyde-3-phosphate dehydrogenase (GAPDH) mRNA was used as an internal control.

**Table 2:** Sequences of primers used for RT-PCR in this study.

Gene Symbol	Accession No.^1^	Sequence (5′ to 3′)^2^	Product (bp)^3^	Cycle	Annealing temp(°C)
Podocalyxin	NM_001018111	F_AAAGGCCAAAAGCTCAGACA	154	29	56
		R_GGACGTTCCCACAACAGTCT			
Endomucin	NM_001159694	F_GAGCGTGAAGCTTCTTACCG	193	26	56
		R_GGTATGGAAGTCGGGATTGA			
GlyCAM-1	NR_003039	F_CTGCTACAGCTCCACCATGA	215	37	56
		R_GTTCTGCAGCTTTCCACCTC			
CD34	NM_001025109	F_GCAAGCCACCAGAGCTATTC	181	29	56
		R_TGCATGTGCAGACTCCTTTC			
CHST2	NM_004267	F_TCCAAGCCTTTCGTGGTATC	209	29	56
		R_GGGAACCCCTTTTAGAGACG			
CHST4	NM_001166395	F_TGGCCATCTTGGCTCTATTC	203	29	56
		R_CTGCTTGAAGGTCATCCACA			
Nepmucin	NM_001168322	F_GCCTGACCTAGAAGCGTTTG	487	29	60
		R_CCTCAAACTCAAGTGCGACA			
PGR	NM_000926	F_ACATGGTAGCTGTGGGAAGG	323	21	56
		R_TTAGTGTGGTGGCAGCTGAG			
ESRα	NM_000125	F_ACAAGCGCCAGAGAGATGAT	361	23	56
		R_AGGATCTCTAGCCAGGCACA			
GAPDH	NM_001256799	F_ACCACAGTCCATGCCATCAC	480	18	56
		R_TCCACCACCCTGTTGCTGTA			

^1^Accession No. refers to the registered No of each respected mRNA. ^2^F: Forward primer; R: Reverse primer. ^3^Amplicon size in base pairs.

For quantitative analysis, it is necessary to determine the linear correlation window of PCR cycles for each sample. Control experiments were performed to determine the range of PCR cycles over which amplification efficiency remained constant. The amplification for these gene products was done using 30 sec. at 94 °C for denaturation, 30 secs at 65 °C for annealing, and 1 min of extension at 68 °C for 18 to 32 cycles depending on transcript abundance and template complexity, followed by a final extension step at 68 °C for 2 min (Table 2). After an indicated number of PCR cycles (between 18 and 32), 3 μL aliquots of PCR reaction mixtures were collected. PCR products were separated by electrophoresis in 2% agarose gels and ethidium bromide staining. Images of electrophoresis gels were obtained by using a Typhoon 9410 multiple image scanner (GE, Little Chalfont, Buckinghamshire, United Kingdom) with a 610-nm band-pass emission filter. The densities of target bands in the electrophoresis gel were measured and quantified by using ImageQuant software (Amersham Pharmacia Biotech) (see supplementary information).

### Statistical analysis

Because menopausal endometrium lacks the stimulation of sex hormones, we used the endometrial samples of menopausal women as a control. We assume that the LSL genes in menopausal endometrium have basic expression dosages without sex hormone stimulation. The relative fold changes of gene expression between reproductive and menopausal endometrium could represent the expression patterns of LSL genes in the different phases of the menstrual cycle.

The values obtained from each sample were normalized to GAPDH mRNA expression. The data are presented as means ± standard error of the mean. Nonparametric Kruskal-Wallis^35^ one-way analysis of variance with multiple comparisons was performed to examine differences between the phases of the menstrual cycle. Findings with a two-sided P value < 0.05 were considered to indicate statistically significant differences between the phases. All data analysis was performed using SPSS version 10.0 (Chicago, IL, USA).

## Electronic supplementary material

Supplementary Information
