# Supplementary material for: Gene expression of human endometrial L-selectin ligand in relation to the phases of the natural menstrual cycle
Source: Sci Rep. 2018 Jan 23;8:1443. doi: 10.1038/s41598-018-19911-z (PMC5780486; doi:10.1038/s41598-018-19911-z)
Supplement: Supplementary file 1 — Supplementary Information [file 41598_2018_19911_MOESM1_ESM.pdf]

Gene expression of human endometrial L-selectin ligand in relation to the phases of the natural menstrual cycle

Tsung-Hsuan Lai[1, 2, 3], Fung-Wei Chang[4], Jun-Jie Lin[3, 5], and Qing-Dong Ling[3, 5*]

[1] Department of Obstetrics and Gynecology, Cathay General Hospital, Taipei, 10693, Taiwan

[2] School of Medicine, Fu Jen Catholic University, New Taipei City, 24205, Taiwan

[3] Institute of Systems Biology and Bioinformatics, National Central University, Taoyuan City, 32001, Taiwan

[4] Department of Obstetrics and Gynecology, Tri-Service General Hospital, National Defense Medical Center, Taipei, 11490, Taiwan

[5] Cathay Medical Research Institute, Cathay General Hospital, New Taipei City, 22174, Taiwan

Financial support: This work was financially supported by a research grant (CGH-MR-9705) from Cathay General Hospital, Taipei, Taiwan.

*To whom correspondence should be addressed.

Qing-Dong Ling, Ph.D.

Institute of Systems Biology and Bioinformatics, National Central University, Jhongli District, Taoyuan City, Taiwan;

E-mail: qdling@hotmail.com

tslai382@gmail.com

Fax: +886-2-27082121 ext. 3557

Tel: 886-2-27082121 ext. 3557

Supplementary Materials

Supplementary information contains: Supplementary Table, Supplementary Figures and Legends.

Supplementary Table 1: Patient information

Case	Cycle day	Phase of cycle	Age (y)	Diagnosis	Gravidity	Parity	Height (cm)	Weight (kg)	BMI (kg/m^2)
1	7	Proliferative	43	myoma	2	2	162	61	23.2
2	8	Proliferative	48	myoma	5	2	153.5	69	29.3
3	9	Proliferative	47	myoma, double uterine	3	1	157	60	24.3
4	9	Proliferative	55	myoma	3	2	151	67	29.4
5	10	Proliferative	43	myoma	4	2	162	55	21.0
6	11	Proliferative	43	myoma	2	2	162	52	19.8
7	11	Proliferative	42	myoma	2	2	156	65	26.7
8	12	Proliferative	43	myoma	2	2	150	41	18.2
9	13	Proliferative	44	myoma	0	0	162	102	38.9
10	14	Proliferative	39	myoma	2	2	156	48	19.7
11	14	Proliferative	46	myoma	3	2	168	60	21.3
12	15	Early-secretory	45	myoma	2	2	163	52.7	19.8
13	15	Early-secretory	47	myoma	3	1	153	55	23.5
14	16	Early-secretory	46	myoma	3	3	159.5	57	22.4
15	16	Early-secretory	35	myoma	2	2	158	60	24.0
16	17	Early-secretory	46	myoma	2	1	160	55	21.5
17	17	Early-secretory	51	myoma	5	3	157	54	21.9
18	18	Early-secretory	44	myoma	5	2	157	48	19.5
19	18	Early-secretory	51	myoma	3	2	160	56	21.9
20	19	Early-secretory	44	myoma	3	2	164	58	21.6
21	20	Mid-secretory	43	myoma	2	2	151	55	24.1
22	20	Mid-secretory	39	myoma	1	1	158	82	32.8
23	21	Mid-secretory	42	myoma, uterine prolapse	5	3	150	48	21.3
24	21	Mid-secretory	56	myoma	2	2	155	59	24.6
25	21	Mid-secretory	45	myoma	2	2			
26	22	Mid-secretory	41	myoma	2	2	163	57	21.5
27	22	Mid-secretory	51	myoma	3	3	155	55	22.9

28	22	Mid-secretory	49	myoma	5	3	159	62	24.5
29	23	Mid-secretory	49	Carcinoma in situ of cervix, myoma	2	2	163	54	20.3
30	23	Mid-secretory	46	myoma	3	3	150	54	24.0
31	26	Late-secretory	48	myoma	4	3	158	67	26.8
32	26	Late-secretory	52	myoma	2	2	158	67	26.8
33	27	Late-secretory	44	myoma	6	3	152	60	26.0
34	28	Late-secretory	46	myoma	2	2	162	52	19.8
35	28	Late-secretory	52	myoma	3	2	157	60	24.3
36	29	Late-secretory	53	myoma	3	2	164	58	21.6
37	29	Late-secretory	43	myoma	3	3	163	73	27.5
38	29	Late-secretory	46	myoma	2	2	170	85	29.4
39	31	Late-secretory	46	myoma	2	2	168	75	26.6
40	32	Late-secretory	47	myoma	5	2	165	63	23.1
41	34	Late-secretory	50	myoma	3	3	152	58	25.1
42		Menopause	61	uterine prolapse, stress incontinence	4	4	153	60	25.6
43		Menopause	72	uterine prolapse	6	3	154	54	22.8
44		Menopause	58	uterine prolapse	3	2	150	54	24.0
45		Menopause	62	uterine prolapse	2	2	157	50	20.3
46		Menopause	75	uterine prolapse	4	4	152	60	26.0
47		Menopause	54	uterine myoma	2	2	157	54.5	22.1
48		Menopause	59	uterine prolapse	2	2	162	38	14.5
49		Menopause	61	uterine prolapse, stress incontinence	3	2	151	61	26.8
50		Menopause	52	uterine	3	3	160	101	39.5

			prolapse					
51	Menopause	55	uterine prolapse	4	2	163.5	62	23.2
52	Menopause	48	uterine prolapse	3	3	152	45	19.5

There were 41 endometrial biopsies, which included 11 from the proliferative phase (days 7 to 14), 9 from the early-secretory phase (days 15 to 19), 10 from the mid-secretory phase (days 20 to 24), and 11 from the late-secretory phase (days ≧ 25). In addition, 11 endometrial samples were obtained as controls from menopausal women with uterine prolapse who underwent vaginal hysterectomy. The mean ages of cases in the proliferative, early-secretory, mid-secretory, and late-secretory phase were 44.8±4.0, 45.4±4.4, 46.1±4.9, and 47.9±3.2 years, respectively. The average BMI of the cases was 24.7±5.7 kg/m^2 for the proliferative phase, 21.7±1.4 kg/m^2 for the early-secretory phase, 24.0±3.4 kg/m^2 for the mid-secretory phase, and 25.1±2.6 kg/m^2 for the late-secretory phase. The mean age and BMI of the cases were not significantly different among the various phases.

Supplementary Figure 1: Examples of full-length gel electrophoresis of RT-PCR products

1-A

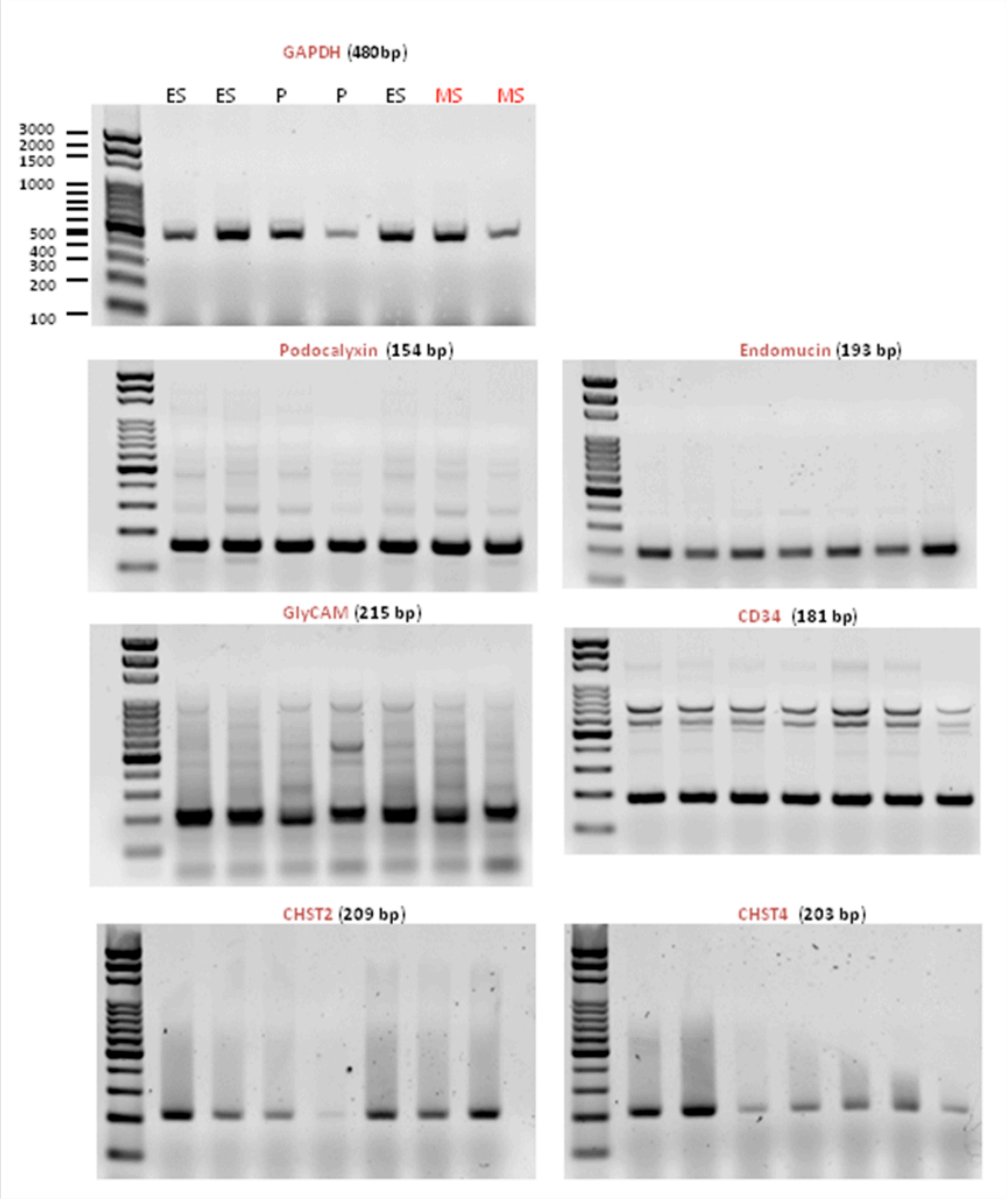

The gel electrophoresis images of RT-PCR products for 7 patients with different phases are presented in Figure 2-A. These 7 patients who exhibited various mRNA concentrations consisted of 3 in ES, 2 in P, and 2 in MS. The mRNA levels of target genes were normalized to GAPDH mRNA levels. The corresponding results obtained from the same samples are labeled in red and indicated in both Figure 2 and Supporting Figure 2-A. (P: proliferative phase; ES: early-secretory phase; MS: mid-secretory phase; LS: late-secretory phase)

1-B

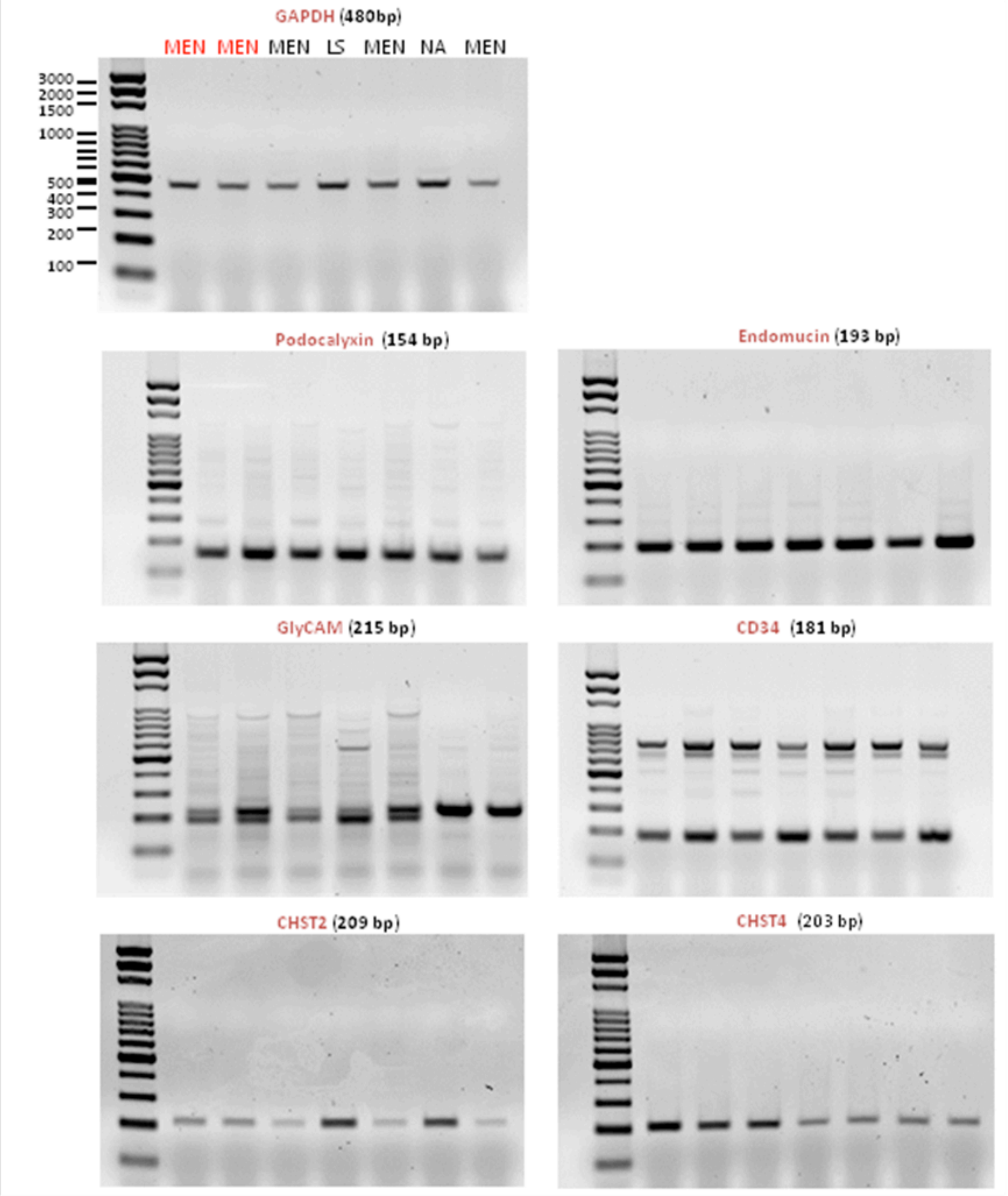

The gel electrophoresis image of RT-PCR products for another 7 patients with different phases (that is different from those in Figure 2-A) are presented in Figure 2-B. These patients, included 5 in MEN, 1 in LS, and 1 in NA, also exhibited different mRNA concentrations. The mRNA levels of target genes were normalized to GAPDH mRNA levels. The corresponding results obtained from the same samples are labeled in red and indicated in both Figure 2 and Supporting Figure 2-B. (LS: late-secretory phase; MEN: Menopause; NA: not available)

Supplementary Figure 2: Comparison of the difference in endomucin expression between the proliferative and early-secretory phases.

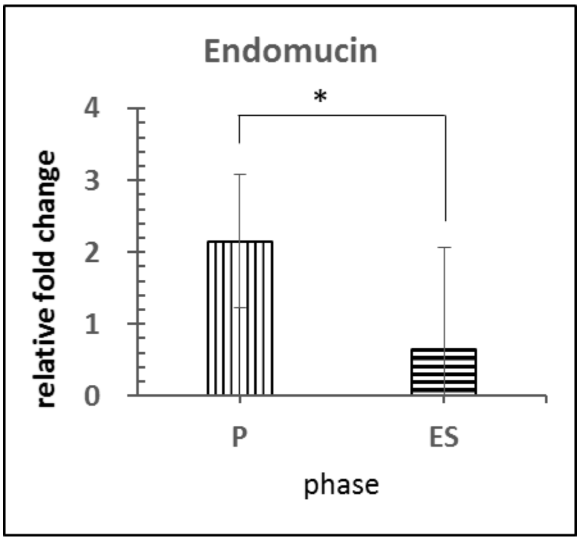

Post-hoc Mann-Whitney U test was performed for comparing the difference in endomucin expression between patients with P and ES phases. The statistical analysis indicated that a significant difference was found between the two groups ($P=0.037 < 0.05$**).** (P: proliferative phase; ES: early-secretory phase)

One Step Reverse-Transcription Polymerase Chain Reaction (RT-PCR)

The RT-PCR in this study was performed with a One Step RT-PCR Kit (BD Biosciences Clonetech, Palo Alto, CA). The amplification of these gene products was performed as: 30 sec at 94℃ for denaturation; 30 sec at 65 ℃ for annealing (different Tms were used depending on different target genes); and 1 min of extension at 68 ℃ (different cycles were used depending on transcript abundance and template complexity); followed by a final extension step at 68℃ for 2 min. The PCR reactions were performed with a Biometra T-Gradient Thermoblock Thermal Cycler Laboratory PCR Detector System (Biometra, Göttingen, Germany).

The PCR products were separated by electrophoresis in 2% agarose gels with ethidium bromide staining.

The completed gels were imaged by a Typhoon 9410 multiple image scanner (GE, Little Chalfont, Buckinghamshire, United Kingdom) that is equipped with a 610-nm band-pass emission filter.

The densities of each target bands in the electrophoresis gel image were measured and quantified by the ImageQuant software (Amersham Pharmacia Biotech). (Ta: annealing temperature; Tm: melting temperature; Tm = 2°C (A+T) + 4°C (G+C)
